# Association between periodontitis treatment and dementia in Taiwanese adults

**DOI:** 10.1186/s12903-023-03551-5

**Published:** 2023-12-06

**Authors:** Hui-Ling Chen, Dai-Rong Wu, Jhih-Jhen Chen, Wei-Szu Lin, I-Chieh Chen, Jeng-Fen Liu, Shine Lien, Ching-Heng Lin

**Affiliations:** 1 Department of Stomatology, Taichung Veterans General Hospital, Taichung, Taiwan; 2 School of Dentistry, National Yang Ming Chiao Tung University, Taipei, Taiwan; 3 Department of Medical Research, Taichung Veterans General Hospital, Taichung, Taiwan; 4 Department of Pediatric Dentistry, Taichung Veterans General Hospital, Taichung, Taiwan; 5 Department of Industrial Engineering and Enterprise Information, Tunghai University, Taichung, Taiwan; 6 Department of Public Health, College of Medicine, Fu Jen Catholic University, New Taipei City, Taiwan; 7 Institute of Public Health and Community Medicine Research Center, National Yang Ming Chiao Tung University, Taipei, Taiwan; 8 Department of Medical Research, China Medical University Hospital, Taichung, Taiwan

**Keywords:** Periodontitis, Dementia, Scaling treatment, Adults

## Abstract

**Background:**

The chronic systemic inflammatory response in periodontitis may be a potential risk factor for dementia, especially in adults. This study determined the association between periodontal treatment and dementia in adults and evaluated the effect of regular scaling treatment on the risk of dementia in this population.

**Methods:**

This case–control study identified 18,930 patients with a dementia-related diagnosis from the Taiwan National Health Insurance Research Database. Scaling and periodontal emergency treatments were evaluated after 1 year and 3 years. Using multivariable logistic regression analysis to evaluate the association between periodontal emergency treatment and dementia risk.

**Results:**

The results showed that scaling treatment rates were lower in the dementia cohort than the non-dementia cohort after 1 and 3 years. Patients who received periodontal emergency treatment within 3 years had a significantly increased risk of dementia. Furthermore, patients with periodontitis who did not receive scaling treatment within 3 years had a higher risk of dementia than patients without periodontitis (OR, 1.22; 95% CI, 1.10–1.35).

**Conclusion:**

This study demonstrated that periodontitis and dementia are associated, and that periodontitis is a risk factor for dementia in adults. The risk of dementia was dependent on the periodontal health status of adults, and our findings suggest that regular scaling can reduce the incidence of dementia in adults. Therefore, regular and routine scaling treatment is suggested for adults.

## Introduction

Periodontitis is a chronic inflammatory condition affecting the periodontium. Periodontitis development is associated with genetic, environmental, and behavioral factors. Moreover, periodontitis may exacerbate several chronic diseases, thereby affecting general health [1]. Periodontitis is characterized by osteoclastogenesis, which results in alveolar bone breakdown and an irreversible loss of support. Moreover, tooth loss is induced by extensive periodontal pockets and progressive attachment loss [2]. A study reported a prevalence rate of 10–60% for periodontitis in adults [3]. Both severe and mild periodontitis are more common in the third and fourth decades of life. A study reported a global frequency of approximately 10% for severe periodontitis [4]. Another study revealed that periodontitis prevalence is influenced by demographic factors, such as age, sex, race, and financial position [5]. Furthermore, periodontitis and tooth loss are associated with various chronic diseases and conditions that negatively affect overall health.

Dementia causes cognitive function loss, thereby making it difficult to perform daily chores and participate in social activities [6]. It is a broad term for mental ability deterioration in individuals; Alzheimer disease (AD) is the most common cause of dementia in adults, although the disease also affects the younger population [7]. Periodontitis and dementia are highly common, especially among adults [7].

AD, which accounts for 60–70% of all dementia cases [8], is a multifactorial neurodegenerative disorder with a complex etiology. According to a study by the United Nations, one in every 85 individuals would have a diagnosis of AD-related dementia by 2050. [9] Moreover, a study reported that dementia prevalence increases exponentially after the age of 65 years, and dementia incidence doubles with every 5.9-year increase in age, from 3.1/1000 person-years for the 60–64 years age group to 175/1000 person-years for the age group of ≥ 95 years [10]. Dementia is mostly caused by aging and is one of the most serious social health issues in the developed world [10].

Several studies have linked dental health to dementia. Kusdhany et al. [11] stressed the link between dental cleanliness and cognitive performance. In Japan, a prospective cohort study revealed that tooth loss is a major risk factor for cognitive impairment in adults [12]. Martande et al. [13] compared periodontal health status between individuals with and without AD and reported that the periodontal health of individuals with AD deteriorated as the condition progressed and was linked to their cognitive performance. Therefore, these studies have discovered a link between poor dental health and dementia; the results may explain the occurrence and prevalence of dementia [14].

Periodontitis and dementia are highly prevalent, especially in adults. Numerous investigations have revealed an association between these two disorders. Several studies have revealed the shared pathophysiological mechanism of these two illnesses in specific research populations. In its severe phase, dementia reduces patients’ quality of life and life expectancy, thereby emotionally and financially affecting patients and their caretakers [15]. Because dementia is caused by various diseases, a single cure for the condition is unlikely [16]. Several studies have revealed the shared pathophysiological mechanism of these two illnesses in specific research populations. In its severe phase, dementia reduces patients’ quality of life and life expectancy, thereby emotionally and financially affecting patients and their caretakers. Because dementia is caused by various diseases, a single cure for the condition is unlikely. Presently, no known “cure” for dementia exists [17]. Thus, to assess the risk factors for dementia, we used epidemiological data. This study evaluated the association between regular periodontal treatment and dementia in a large Taiwanese national sample to gain a better understanding of the disease mechanisms.

## Materials and methods

### Data sources

This retrospective case–control study was conducted using data from the Taiwan National Health Insurance Research Database (NHIRD), which is maintained by the National Health Insurance program. The program, established by Taiwan’s Ministry of Health and Welfare on March 1, 1995, covers 99.9% of the Taiwanese population and collects information in a complete and standardized format to meet researchers’ requirements in different fields [18]. The National Health Insurance Administration regularly audits original medical records to improve the accuracy of the claims data. As the NHIRD provides identified and anonymous claims data to the academic community for epidemiological studies, the informed consent requirement was waived. This research project was approved by the Institutional Review Board of Taichung Veterans General Hospital (IRB no. CE19166A-2), and this study was conducted in accordance with the principles of the Declaration of Helsinki. The NHIRD provided all available data. Because the claims database used in this study solely contained deidentified data, informed consent was not required. The Strengthening the Reporting of Observational Studies in Epidemiology reporting guidelines were followed in this study.

### Study design

**Fig. 1:**
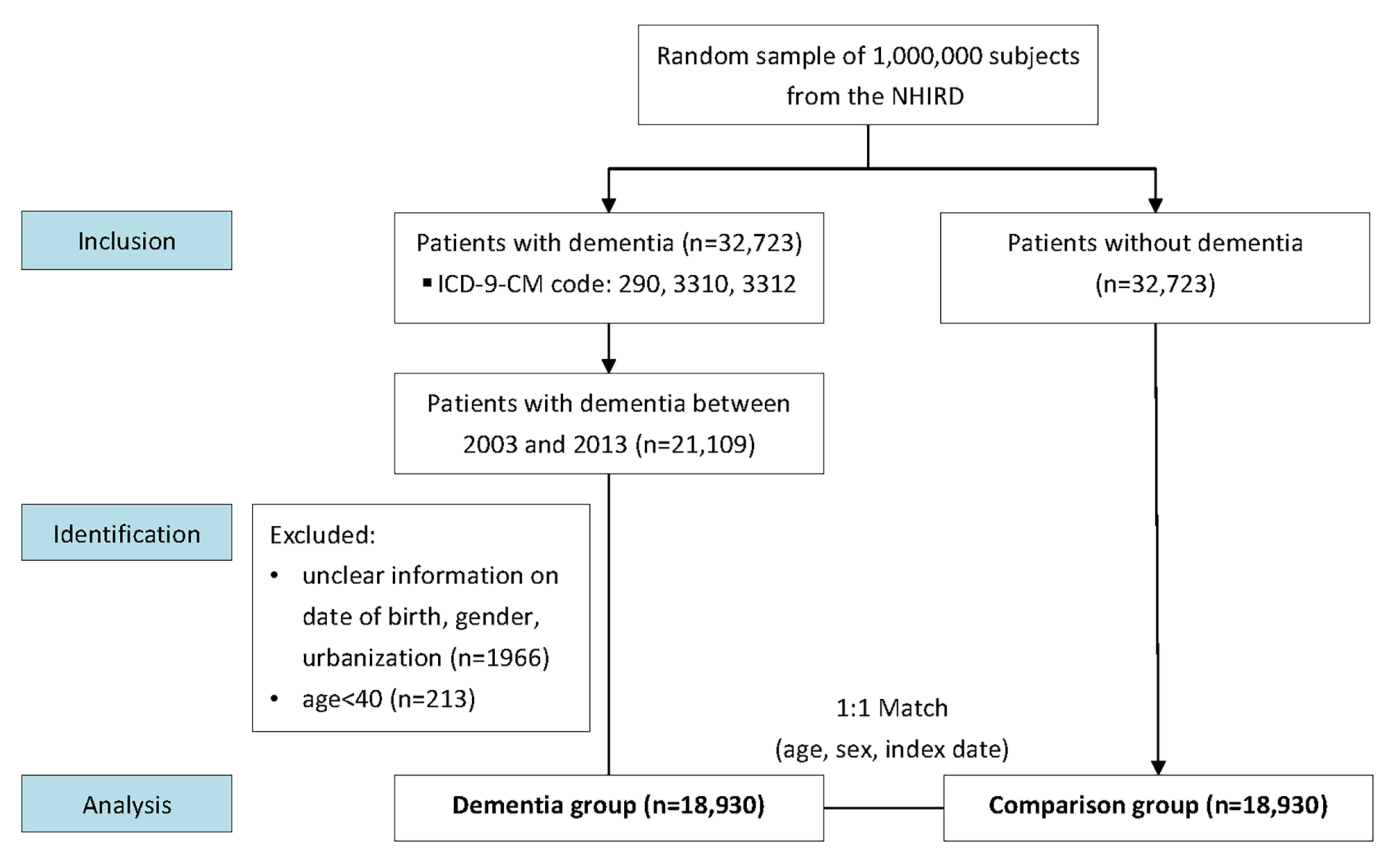
STROBE flow diagram: flowchart illustrating of the study design

We selected 32,723 patients aged 40 years and more with a dementia-related diagnosis (International Classification of Diseases, 9th Revision, Clinical Modification [ICD-9-CM] codes 290, 3310, and 3312) between January 1, 2003, and December 31, 2013 (Fig. 1). The index date was defined as the first-time of dementia-related diagnosis between 2003 and 2013, recorded at least thrice during outpatient visits or once during hospitalization. A total of 13,793 patients with incomplete data on birth, sex, and residential urbanization level during the study period were excluded. The final study population consisted of 18,930 patients in the dementia cohort; the nondementia cohort, consisting of 18,930 individuals without dementia, was matched to the dementia cohort according to age, sex, and index date at a ratio of 1:1. Because periodontitis and dementia are associated, we confirmed scaling and periodontal emergency treatments before and after dementia-related diagnosis.

### Covariates

Age, sex, residential urbanization level, delivery model, and comorbidities were used as factors in our study. The urbanization level in Taiwan was classified into three categories: urban, suburban, and rural areas. This categorization was based on a combination of factors, including population density, the proportion of residents with higher education, the elderly population, the agricultural population, and the number of physicians per 100,000 people [19]. ICD-9-CM codes were used to classify diagnosis and procedures. Diabetes mellitus (ICD-9-CM code 250), hypertension (ICD-9-CM codes 401–405), and hyperlipidemia (ICD-9-CM code 272) were recorded. Scaling is a preventive and routine treatment to remove dental calculus from teeth surfaces to prevent the development of periodontal diseases. Periodontal emergency treatments are interventions taken in acute situations to alleviate symptoms, control infections, and safeguard the health of teeth and periodontal tissues. Periodontal emergency treatment can be identified with the NHI procedure code 91,001 C. Scaling, whether localized or full mouth, corresponds to the health insurance procedure codes 91,003 and 91,004 C, respectively.

Emergency periodontal treatments hold significant importance within the realm of dental care, as they offer immediate symptom relief, effective infection management, complication prevention, and the preservation of both teeth and overall oral well-being [20]. This intervention not only curtail the spread of infections but also ensure patient comfort and uphold aesthetic integrity.

The delivery model employed in this study defines the identification of specific diagnostic codes based on their usage frequency. Specifically, diagnostic codes were considered for inclusion if they were used at least once during hospital admission or at least three times in the outpatient service.

### Statistical analysis

SAS 9.4 (SAS Institute Inc., Cary, NC, USA) was used to conduct all statistical analyses. Differences in the distribution of characteristics between the dementia and nondementia cohorts were examined using the chi-squared test, as appropriate. To examine the association between periodontal emergency treatment and dementia risk, univariable and multivariable logistic regression models were used. Odds ratios (ORs) and 95% confidence intervals (95% CIs) were calculated. The regression models were adjusted for the number of medical visits during the 3 years before the index date. The association between periodontal emergency treatment and dementia was determined, and the study was stratified by age group. All statistical tests were two-tailed, and *p* values of less than 0.05 were deemed statistically significant.

## Results

A total of 18,930 patients with a dementia-related diagnosis who were aged 40 years and more were included. The demographic characteristics, including age, sex, residential urbanization level, comorbidities, and procedures, are listed in Table 1. Participants were stratified into distinct age groups to comprehensively analyze our sample, accounting for age-related variations. The age distribution is as follows: 40–59 (7.5%), 60–69 (14.9%), 70–79 (37.4%), 80–89 (34%), and 90+ (6.2%). Compared with patients who received regular scaling treatment, patients who did not receive regular scaling treatment exhibited a significantly higher risk of dementia. The scaling treatment rate in the dementia cohort was lower, with the rates of 18.3% and 34.8% after 1 year and 3 years, respectively. In the nondementia cohort, the scaling treatment rate was 19.6% and 36.4% after 1 year and 3 years, respectively. The dementia cohort underwent a greater number of periodontal emergency treatments during the follow-up period (1 year and 3 years). A higher proportion of diabetes mellitus, hypertension, and hyperlipidemia cases was observed in the dementia cohort than in the nondementia cohort (*p* < 0.001). Moreover, no significant difference in the residential urbanization level and periodontal treatment procedure was observed between the dementia and nondementia cohorts.

**Table 1:** Characteristics of study subjects

Characteristic	Comparison group	Dementia group	Total	*P*-value
	(n = 18,930)	(n = 18,930)		
	n (%)	n (%)	n	
**Age**				1.000
40–59	1427 (7.5)	1427 (7.5)	2854	
60–69	2828 (14.9)	2828 (14.9)	5656	
70–79	7071 (37.4)	7071 (37.4)	14,142	
80–89	6429 (34)	6429 (34)	12,858	
90+	1175 (6.2)	1175 (6.2)	2350	
**Gender**				1.000
Female	9889 (52.2)	9889 (52.2)	19,778	
Male	9041 (47.8)	9041 (47.8)	18,082	
**Urbanization**				0.161
Urban	10,540 (55.7)	10,412 (55)	20,952	
Suburban	2787 (14.7)	2915 (15.4)	5702	
Rural	5603 (29.6)	5603 (29.6)	11,206	
**Comorbidity**				
Diabetes mellitus	3530 (18.6)	5165 (27.3)	8695	< 0.001
Hypertension	9047 (47.8)	11,048 (58.4)	20,095	< 0.001
Hyperlipidemia	3263 (17.2)	3750 (19.8)	7013	< 0.001
**Procedures (before 1 year)**				
Periodontal emergency treatment	1461 (7.7)	1526 (8.1)	2987	0.215
Scaling localized/ full mouth	3704 (19.6)	3464 (18.3)	7168	0.002
Comprehensive periodontal treatment	7 (0)	9 (0)	16	0.617
**Procedures (before 3 years)**				
Periodontal emergency treatment	3107 (16.4)	3325 (17.6)	6432	0.003
Scaling localized/ full mouth	6887 (36.4)	6596 (34.8)	13,483	0.002
Comprehensive periodontal treatment	13 (0.1)	13 (0.1)	26	1.000

^a^ Comparison of categorical variables were analyzed using the Chi-square test

Table 2 lists the results of multivariable logistic regression analyses of the association between periodontal emergency treatment (localized or full mouth scaling) and dementia in each cohort. The OR for Model 1 was 1.06 (95% CI, 1.00–1.12), indicating that patients who received periodontal emergency treatment within 3 years exhibited a significant increase in the risk of dementia. The OR for Model 2 was 0.95, indicating that patients who received scaling treatment within 3 years exhibited a low risk of dementia (95% CI, 0.91–0.99). The OR for Model 3, which was adjusted for all confounding factors, was 1.09 (95% CI, 1.03–1.16) for the periodontal emergency treatment cohort and 0.92 (95% CI, 0.88–0.97) for the scaling treatment cohort; ORs for all groups were statistically significant (*p* < 0.001). These results indicated that patients who received scaling treatment within 3 years exhibited a lower risk of dementia than those who did not receive scaling treatment within 3 years. After adjustment for patients with periodontal emergency treatment, who are likely to have more severe periodontitis, the risk of dementia was lower in patients who received scaling treatment than in those who did not receive scaling treatment (Table 2).

**Table 2:** Multivariable analysis of factors associated with dementia

Variables	Model 1				Model 2				Model 3			
	OR	95%CI		P-value	OR	95% CI		P-value	OR	95% CI		P-value
**Procedures (before 3 years)**												
Periodontal emergency treatment	1.06	1.00	1.12	0.048					1.09	1.03	1.16	0.003
Scaling localized/ full mouth					0.95	0.91	0.99	0.015	0.92	0.88	0.97	0.001
**Age**												
40–59	1.00(reference)				1.00(reference)				1.00(reference)			
60–69	0.87	0.79	0.95	0.003	0.87	0.79	0.95	0.002	0.87	0.79	0.95	0.002
70–79	0.83	0.76	0.90	< 0.001	0.82	0.76	0.89	< 0.001	0.82	0.76	0.89	< 0.001
80–89	0.84	0.77	0.91	< 0.001	0.83	0.76	0.90	< 0.001	0.83	0.76	0.90	< 0.001
90+	0.91	0.81	1.02	0.092	0.89	0.79	0.99	0.036	0.89	0.79	0.99	0.036
**Gender**												
Female	1.00(reference)				1.00(reference)				1.00(reference)			
Male	1.03	0.99	1.07	0.170	1.03	0.99	1.07	0.155	1.03	0.99	1.07	0.161
**Urbanization**												
Urban	1.00(reference)				1.00(reference)				1.00(reference)			
Suburban	1.08	1.02	1.14	0.015	1.07	1.01	1.13	0.035	1.07	1.01	1.13	0.029
Rural	1.04	0.99	1.09	0.114	1.03	0.98	1.08	0.258	1.03	0.98	1.08	0.223
**Comorbidity**												
Diabetes mellitus	1.53	1.45	1.61	< 0.001	1.52	1.45	1.60	< 0.001	1.52	1.45	1.60	< 0.001
Hypertension	1.44	1.38	1.50	< 0.001	1.44	1.38	1.51	< 0.001	1.44	1.38	1.50	< 0.001
Hyperlipidemia	0.95	0.90	1.00	0.055	0.95	0.90	1.01	0.098	0.95	0.90	1.01	0.082

**Fig. 2:**
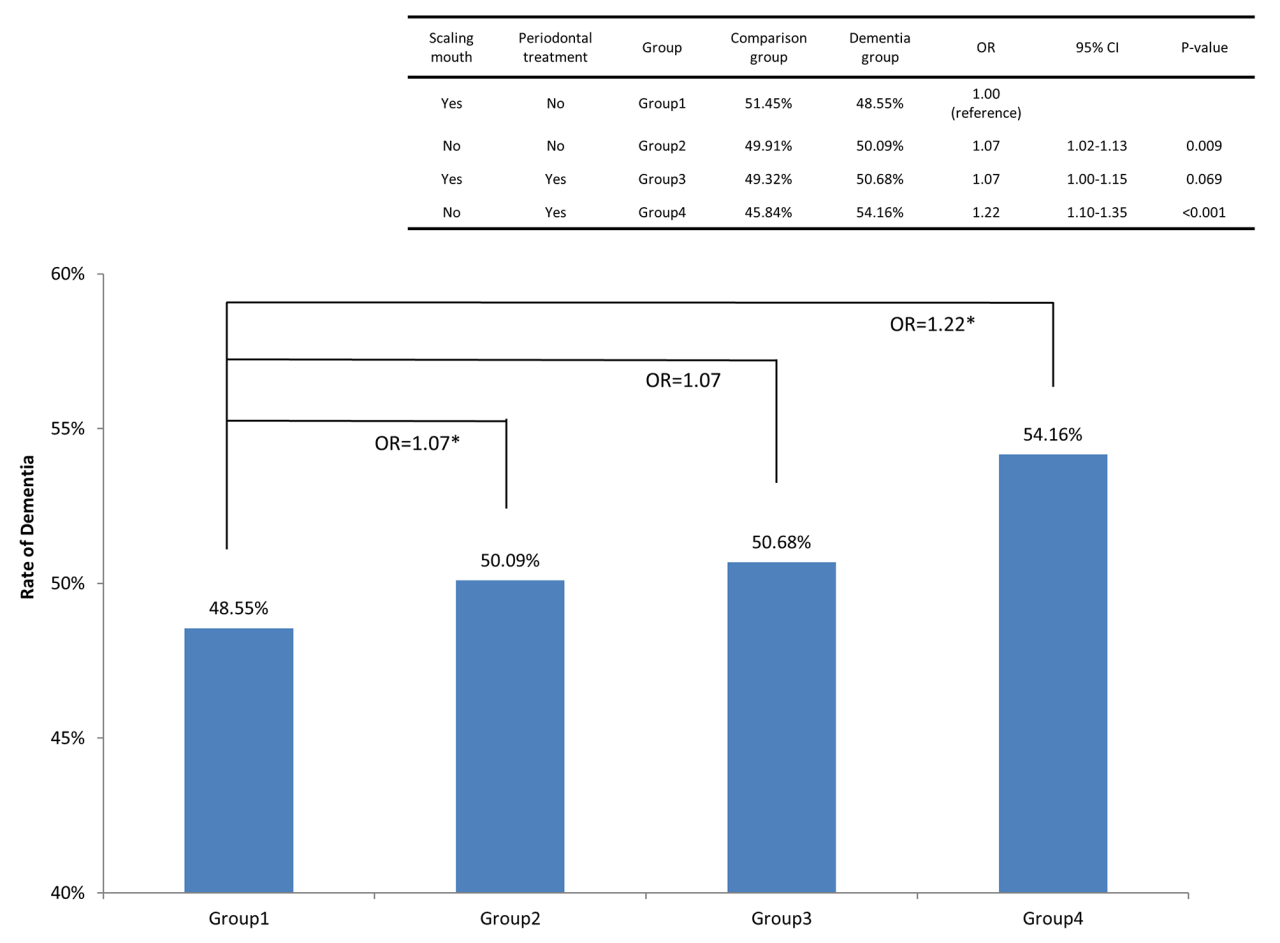
Ratios of dementia among different periodontal treatments. We divided the study patients into four groups according to periodontitis diagnosis and whether they scaling treatment

Because periodontitis is a major cause of dementia, we divided the study patients into four groups according to periodontitis diagnosis and whether they scaling treatment. The results revealed that patients with periodontitis who did not receive scaling treatment within 3 years exhibited a higher risk of dementia (OR, 1.22; 95% CI, 1.10–1.35). Compared with patients without periodontitis and who received scaling treatment within 3 years, the risk of dementia increased by 22% in patients with periodontitis who did not receive scaling treatment within 3 years (Fig. 2).

## Discussion

This case–control study demonstrated a significant association between periodontitis and dementia in adults. Adults who did not receive scaling treatment within 3 years exhibited a significantly higher risk of dementia than those who received the treatment within 3 years. Therefore, regular dental visits for scaling and oral hygiene instructions are effective in reinforcing the masticatory muscle and consequently reducing dementia risk in adults.

Dementia develops slowly over 5–20 years after commencement; therefore, dementia prevention is crucial [21]. In this study, age, sex, and residential urbanization level were explored as sociodemographic variables, whereas diabetes, hypertension, and hyperlipidemia were investigated as comorbidities. The major risk factor for dementia is old age. In this study, dementia prevalence was 37.4% in patients aged 70–79 years and 34% in patients aged 80–89 years; the results are consistent with those of a previous study [10]. Moreover, the risk of dementia was significantly higher in women than in men, a result consistent with that of a previous study [22]. Diabetes mellitus, hypertension, and hyperlipidemia are also risk factors for dementia [23, 24], and the prevalence of these diseases increases with age. Our study revealed a higher prevalence of dementia in patients with diabetes mellitus, hypertension, or hyperlipidemia (*p* < 0.001) than in patients without these comorbidities. Therefore, preventing chronic illnesses with regular health assessments before the onset of old age may reduce the risk of dementia.

Emergency periodontal treatments hold significant importance within the realm of dental care, as they offer immediate symptom relief, effective infection management, complication prevention, and the preservation of both teeth and overall oral well-being [20]. This intervention not only curtail the spread of infections but also ensure patient comfort and uphold aesthetic integrity. Periodontitis is a serious infection of the gums and elicits a chronic inflammatory response. Studies have linked periodontitis to heart disease, diabetes, hypertension, respiratory disease, osteoporosis, metabolic syndrome, and rheumatoid arthritis [25, 26]. Moreover, recent studies have revealed the association between periodontitis and dementia [27, 28]. These findings were similarly to Seulggie Choi et al. reported that in comparison to individuals without chronic periodontitis, patients afflicted with chronic periodontitis exhibited a heightened susceptibility to general dementia (adjusted hazard ratio [aHR] = 1.06; 95% confidence interval [CI] = 1.01–1.11) and Alzheimer’s disease (aHR = 1.05; 95% CI = 1.00-1.11) [29]. Additionally, Periodontitis may lead to immunoinflammatory response activation [1]. Periodontitis can activate the immune system, resulting in inflammatory reactions. Free radicals and toxic chemicals produced during inflammatory reactions cause cell damage and directly or indirectly cause dementia [27, 30]. Sparks et al. revealed that periodontitis is a risk factor for the onset and progression of AD [31]. In addition, a study reported an exacerbated inflammatory condition in adults, which accelerates neurodegenerative disease progression [28]. Furthermore, some studies have postulated several pathophysiological processes that may explain the role of periodontitis in AD etiology. The transfer of pathogens and inflammatory mediators from the mouth to the systemic circulation is one of the processes. Patients may develop bacteremia when the physical, chemical, and immunological barriers of the oral cavity are compromised [32, 33]. Lipopolysaccharides can accumulate in gingival tissue, thereby initiating and prolonging local inflammatory reactions by increasing proinflammatory cytokine production; they also play a vital role in specific cell death processes by translocating between the stomach, blood, and other tissues [32, 33]. Bacteria and proinflammatory cytokines from inflamed periodontal tissue can reach the circulation, causing or worsening inflammation and increasing the levels of prostaglandins and cytokines, including interleukin-1 (IL-1), IL-6, and tumor necrosis factor [34–36]. Periodontal infections and their products increase the production of proinflammatory cytokines, including IL-1, IL-6, and tumor necrosis factor. During regular activities like biting, brushing, and flossing teeth, these cytokines are exposed to bacteria repeatedly; the cytokine receptors become saturated, and they enter the systemic circulation [37], causing inflammation.

According to another pathophysiological process that may link periodontitis and AD, microorganisms from dental plaque may gain entry into the brain tissue through direct tissue invasion, blood flow, or peripheral nerves. *Aggregatibacter actinomycetemcomitans*, *Porphyromonas gingivalis*, *Tannerella forsythia*, *Fusobacterium nucleatum*, and *Prevotella intermedia* have been reported to be associated with brain abscess, suggesting their ability to penetrate the brain tissues. A study reported the presence of several oral *Treponema* species, such as *Treponema denticola*, more frequently in the brains of patients with AD than in healthy controls [38]. However, limited evidence indicates that gingivitis and impaired masticatory performance, in the absence of frequent teeth cleaning, are risk factors for dementia. Moreover, no evidence of a direct effect on caries or periodontitis exists. More dental visits for scaling can lead to better tooth longevity and masticatory function, thereby decreasing the risk of dementia.

The process of confirming a dementia diagnosis typically involves conducting a thorough assessment that includes the exclusion of other potential causes of cognitive impairment. Moreover, it often demands long-term observation and continuous evaluation to monitor the condition’s progression. Regarding Alzheimer’s Disease (AD), which accounts for 60–70% of all dementia cases [8], it is essential to emphasize that AD is a multifactorial neurodegenerative disorder with a complex etiology. Therefore, the need for long-term observation remains paramount in understanding this condition.

Although it has not been proven that poor dental health causes dementia, substantial evidence indicates that oral health deteriorates with the progression of cognitive impairment and dementia. Therefore, dental care must be included in the overall care plan for people with dementia to improve their oral health and quality of life. Moreover, better oral health recommendations and specialized care should be implemented at various stages of dementia. Further studies are required to clarify the association between dental health and dementia, and the results may help to determine whether a high inflammatory load affects dementia progression or development.

Knowledge of oral health is a fundamental prerequisite for healthy behavior, enabling individuals to take measures to protect their own health. The current study suggests that dental care-seeking behavior and oral hygiene practices consistently demonstrate patterns over time, whether assessed at one-year or three-year intervals [39, 40]. Additionally, the findings indicate a consistent approach to treating periodontitis. In other words, individuals tend to maintain similar dental care-seeking behaviors, oral hygiene practices, and periodontitis treatment over both short-term and long-term periods. These results imply stability and consistency in oral health behaviors and treatment patterns within the study population.

This study has some strengths. First, we used an administrative database to perform this population-based cohort study; therefore, we avoided selection bias. However, previous research has relied heavily on personal experiences or medical record analyses from a small number of institutions. The outcome of our trial was determined by physicians’ diagnoses. Therefore, we cannot entirely rule out substantial selection bias. Second, the NHIRD provides complete information on outpatient, inpatient, and prescription visits, ensuring that medical visits were not underreported. Third, because the Han Chinese ethnic group accounts for more than 98% of the population in Taiwan, race was not a factor. Scaling can reduce the risk of AD in adults. Therefore, we suggest that routine periodontal treatment and oral hygiene instructions should be provided to adults every 6 months. A study reported that significantly more patients with dementia required help in brushing their teeth than individuals without dementia; this result supports our suggestion.

However, this study has several limitations. First, the results may have been underestimated because of the absence of some patients’ follow-up data. Second, the data from the NHIRD may not apply to the entire Asian population, because Taiwan is a developed country with a well-established health insurance system. Third, this study did not exclude individuals with potential confounding factors like smoking or those who had received periodontal treatment, which could have led to pre-existing deep periodontal pockets or significant alveolar bone loss. These factors may have influenced the study’s outcomes. Nevertheless, despite these limitations, we could control for recall bias, which is common in questionnaire-based research; this was because of the use of NHIRD data, through which we could confirm the association between dental health and dementia in adults.

## Conclusions

We conducted a retrospective case–control study using a population-based biomedical research database of Taiwanese individuals with Han Chinese ancestry and determined that regular periodontal treatment is proven to lower the risk of dementia. The risk of dementia was markedly increased by the periodontal health status of adults. Our findings suggest that regular scaling can reduce the incidence of dementia. However, prospective studies are required to evaluate the effects of regular scaling treatment on the risk of dementia in adults.

## Data Availability

All data generated or analyzed during this study are included in this published article. The datasets used and analyzed during the current study available from the corresponding author on reasonable request.
